# Port-site metastases after diagnostic laparoscopy in advanced ovarian cancer: a case report and a systematic review of the literature

**DOI:** 10.3389/fsurg.2026.1777548

**Published:** 2026-05-08

**Authors:** Orazio De Tommasi, Sofia Bigardi, Giosuè Giordano Incognito, Linda Modena, Chiara Goretti, Giulia Spagnol, Carla Ettore, Giuseppe Ettore, Marco Noventa, Carlo Saccardi, Roberto Tozzi

**Affiliations:** 1 Department of Women and Children’s Health, Clinic of Gynecology and Obstetrics, University of Padua, Padua, Italy; 2 Obstetrics and Gynecology Unit, Maternal Child Department, ARNAS Garibaldi Nesima, Catania, Italy

**Keywords:** chemotherapy, HGSC (high grade serous ovarian cancer), laparoscopy, port site metastases, trocar site metastases

## Abstract

**Background:**

Diagnostic laparoscopy is widely used in advanced ovarian cancer to assess resectability and avoid futile laparotomies. However, concerns persist regarding the risk of port-site metastases (PSM), a complication that remains poorly characterized, particularly in terms of clinical impact and management.

**Methods:**

We report a case of late-onset giant port-site metastasis occurring after diagnostic laparoscopy in a patient with advanced high-grade serous ovarian cancer. In addition, we conducted a systematic review of the literature according to PRISMA guidelines, including studies reporting PSM after laparoscopy in advanced ovarian cancer. Data on incidence, risk factors, surgical techniques, oncologic outcomes, and management strategies were extracted and analyzed.

**Results:**

The reported case illustrates an uncommon but clinically challenging presentation of delayed, rapidly progressive PSM that ultimately precluded surgical management. The systematic review included five eligible studies and revealed a wide variability in PSM incidence, ranging from 1.18% to 46.7%, depending on detection method and study design. Advanced FIGO stage, large-volume ascites, extensive peritoneal disease, and institutional expertise emerged as the main risk factors. Importantly, PSM did not appear to independently affect overall survival, acting instead as a surrogate marker of aggressive disease biology. Routine port-site resection achieved effective local control but was associated with a significantly increased risk of wound-related morbidity, without a demonstrated survival benefit.

**Conclusions:**

PSM following diagnostic laparoscopy in advanced ovarian cancer are more frequent than clinically appreciated when histologically assessed but do not appear to adversely influence prognosis beyond underlying disease burden. Routine port-site excision should be carefully weighed against its associated morbidity. Diagnostic laparoscopy remains an oncologically safe and cost-effective tool when appropriately performed in specialized centers, and its benefits outweigh the risks when integrated into a standardized surgical pathway.

## 1 Introduction

Ovarian cancer is the most lethal of all gynecological malignancies since more than 75% of affected patients are diagnosed at an advanced stage (1). The standard treatment consists of upfront surgery with the intent to perform maximal cytoreduction, followed by platinum/taxane-based chemotherapy in most cases (2). Residual tumor after surgery is indeed the most significant independent prognostic factor and achieving a complete removal of the tumor is linked to the most favorable survival outcome. In patients where primary cytoreduction is not feasible, the gold standard treatment consists of neoadjuvant chemotherapy (3 or 4 cycles) followed by interval surgery with the goal of achieving extensive tumor reduction (3).

Over the past two decades, exploratory laparoscopy has demonstrated remarkable success in determining preoperatively the extent of the disease avoiding unnecessary laparotomies in patients amenable to neoadjuvant treatment (4). However, diagnostic laparoscopy is a surgical procedure burdened with a series of potential intraoperative complications (such as visceral and vascular injuries) and postoperative complications (wound infections, adhesions, formation of hernias etc*.*) (5).

Among the most dreaded postoperative complications, trocar site metastases must be considered. Laparoscopic port-site metastasis (PSMs) refers to the early recurrence of tumor lesions that develop locally in the abdominal wall within the scar tissue of one or more trocar insertion sites due to iatrogenic disease spread (6). These metastases typically present as subcutaneous nodules located at or near the incision points used for laparoscopic access. Their occurrence raises concern about iatrogenic dissemination of tumor cells, especially in the setting of intraperitoneal malignancies such as epithelial ovarian cancer. PSMs may result from direct tumor inoculation, aerosolization of malignant cells or contamination during instrument exchange or specimen extraction (7).

In the present article, we aim to report a case of late-onset PSM and to perform a systematic review of the literature.

## 2 Case report

We report the case of a 79-year-old woman in whom a contrast-enhanced total-body CT scan revealed, in the pelvic cavity, a solid mass measuring approximately 12 × 8 × 8 cm with inhomogeneous enhancement, likely of adnexal origin, causing distortion of the regional anatomy. Posteriorly, the lesion showed no clear cleavage planes with the rectal wall, while anteriorly it caused a mild impression on the posterior profile of the urinary bladder. A large amount of ascites was present both above and below the mesocolon, involving all abdominal quadrants, associated with nodular thickening of the peritoneum and solid peritoneal implants. Serum CA-125 level was 3025 kU/L.

**Figure 1:**
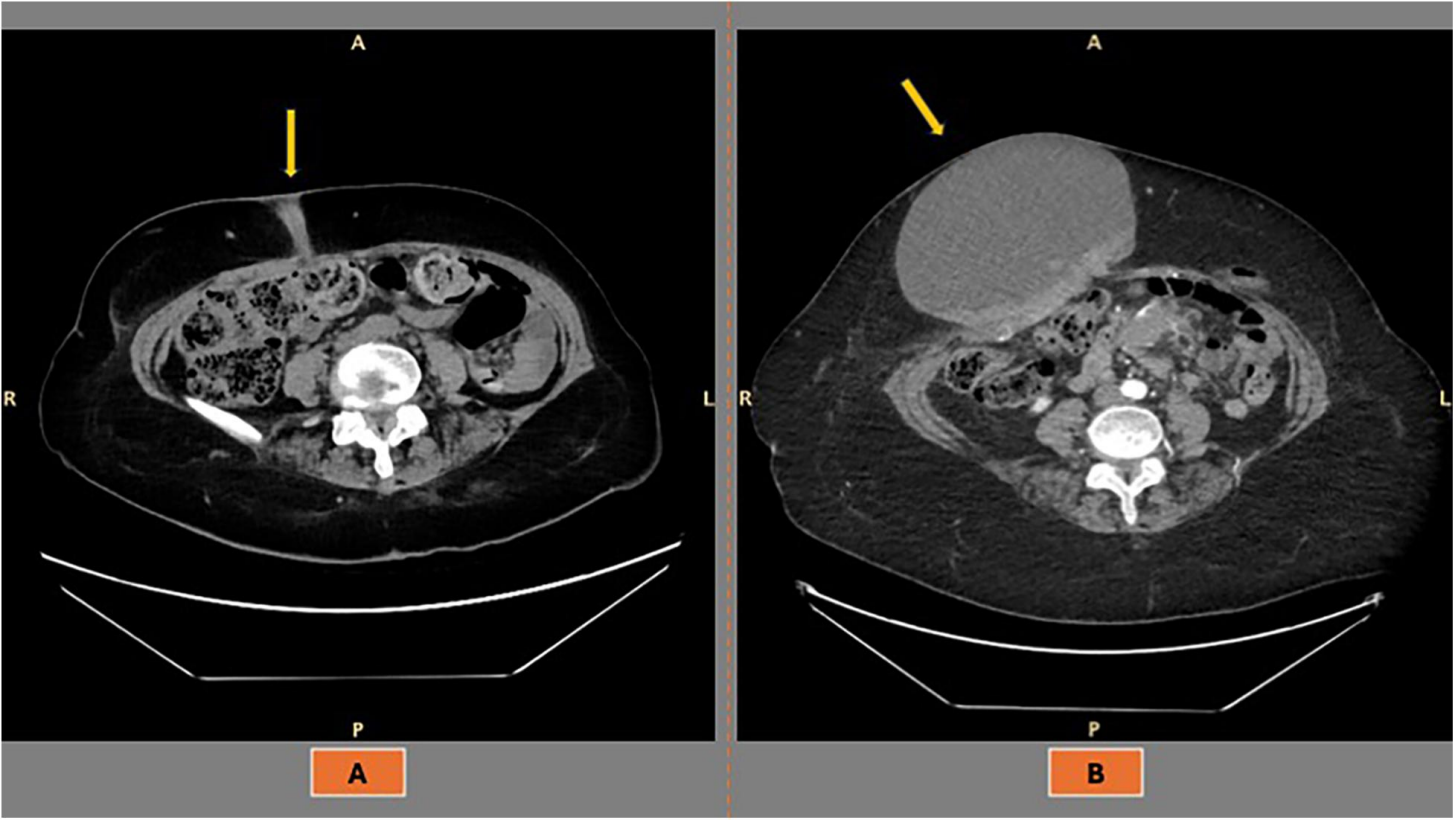
**A**: recurrence of ovarian cancer at the port site, presenting at CT scan as a small lesion in the subcutaneous tissue of the anterior abdominal wall, extending from the skin to the underlying muscle. **B**: The previously described lesion progressed into a giant metastatic mass.

The patient underwent an exploratory laparoscopy in February 2022, which confirmed the presence of peritoneal carcinomatosis and a frozen pelvis, establishing her eligibility for primary cytoreductive surgery. Histological examination of a peritoneal biopsy revealed a high-grade serous ovarian carcinoma, BRCA wild-type and HRD-negative (GIS 22). Cytoreductive surgery was performed one week later and included total abdominal hysterectomy with bilateral salpingo-oophorectomy, sigmoid–rectal resection, extensive peritonectomy of the right diaphragmatic recess, and total omentectomy. Pathological staging was FIGO stage IIIC, with no residual disease. The patient subsequently received adjuvant chemotherapy with carboplatin (AUC 2) plus weekly paclitaxel. She was then placed under surveillance without maintenance therapy due to her fair general condition. In February 2023, a CT scan revealed peritoneal disease recurrence with a small transmural lesion of the anterior abdominal wall, adjacent to a previous trocar site (Figure 1A). Despite systemic chemotherapy initiated at the time of recurrence, the patient developed a large abdominal mass (15 × 18 cm) located between the mesogastrium and the right iliac fossa, consistent with a giant PSM (Figure 1B).

Due to the patient's multiple comorbidities, overall poor performance status, and the inoperability of the abdominal wall lesion, as confirmed by a multidisciplinary consultation involving both plastic and general surgeons, the decision was made to initiate palliative care.

### 2.1 Surgical technique

Exploratory laparoscopy was performed using an open (Hasson) direct-entry technique. The camera trocar was inserted in a controlled fashion through a 1.5-cm infraumbilical incision or, alternatively, at Palmer's point, located 2 cm below the left costal margin along the midclavicular line. The abdominal wall was dissected layer by layer under direct vision, and a Hasson trocar was introduced for placement of the laparoscope. Following establishment of the pneumoperitoneum, three additional ancillary trocars were positioned in the right and left iliac fossae and/or in the suprapubic region to allow proper manipulation of the viscera and adequate surgical exposure. The primary aim of the staging laparoscopy was to assess the presence of three surgical contraindications to primary cytoreductive surgery: small-bowel mesenteric retraction, diffuse miliary serosal involvement of the small intestine, and encasement of the hepatic hilum. Multiple peritoneal biopsies were obtained for histopathological examination. All specimens were retrieved using an Endopouch specimen retrieval system. Pneumoperitoneum was maintained at a standard intra-abdominal pressure of 10–12 mmHg throughout the procedure. At the conclusion of the operation, carbon dioxide was completely evacuated prior to trocar removal. Trocar sites were closed meticulously, with absorbable fascial sutures placed at the camera port site. The total operative time was 26 min.

## 3 Systematic review

A protocol was built *a priori* to systematically review the literature and was registered in the PROSPERO International Prospective Register of Systematic Reviews (ID: CRD420251252088). Each review stage was independently performed by two authors, and disagreements were solved by discussion with a third author. The whole study was reported following the Preferred Reporting Item for Systematic Reviews and Meta-analyses (PRISMA) statement and checklist (8).

### 3.1 Search strategy

A comprehensive literature search was conducted in MEDLINE, Web of Science, and EMBASE from database inception to July 2025. Multiple combinations of topic-related keywords (e.g., *metastas*; recurren*; port; ovarian*; cancer; tumor; carcinoma; neoplasia; malignancy*) were used to identify peer-reviewed studies reporting PSM in women with advanced ovarian cancer. Studies not published in English and review articles were excluded *a priori*.

The electronic search strategy incorporated controlled vocabulary and free-text terms tailored to each database. For MEDLINE, the following search string was applied: [(“port-site metastasis” OR “port site metastasis” OR “port-site recurrence” OR “port site recurrence” OR “trocar-site metastasis” OR “trocar site metastasis” OR “trocar-site recurrence” OR “trocar site recurrence” OR “wound metastasis” OR “wound recurrence” OR “laparoscopic metastasis” OR “laparoscopic recurrence”) [Title/Abstract]) AND [“ovarian cancer” OR “ovarian neoplasms” (MeSH) OR “ovarian carcinoma” OR “ovarian malignancy”]. For EMBASE, the adapted syntax was: (‘port site metastasis':ti,ab OR ‘port-site metastasis':ti,ab OR ‘port site recurrence':ti,ab OR ‘port-site recurrence':ti,ab OR ‘trocar site metastasis':ti,ab OR ‘trocar-site metastasis':ti,ab OR ‘trocar site recurrence':ti,ab OR ‘trocar-site recurrence':ti,ab OR ‘wound metastasis':ti,ab OR ‘laparoscopic metastasis':ti,ab) AND (‘ovarian cancer'/exp OR ‘ovarian neoplasm':ti,ab OR ‘ovarian carcinoma':ti,ab OR ‘ovarian malignancy':ti,ab**)**.

Data from eligible studies were extracted in their original form without modification. Variables of interest included study characteristics (setting, design, study period, sample size), surgical management (approach, staging procedures, number of ports, specimen extraction routes, lymph node removal technique, uterine manipulation, preventive measures against PSM, adjuvant therapy), and primary tumor features (histotype, FIGO grade and stage).

PSM-specific data included: interval from surgery to PSM, number and anatomical sites of PSMs, lesion size, presence of synchronous metastases, metastases preceding PSM, post-PSM recurrence, local PSM management, systemic therapy, disease-free survival after PSM, overall survival from PSM detection, and date of last disease-free follow-up.

## 4 Results

**Figure 2:**
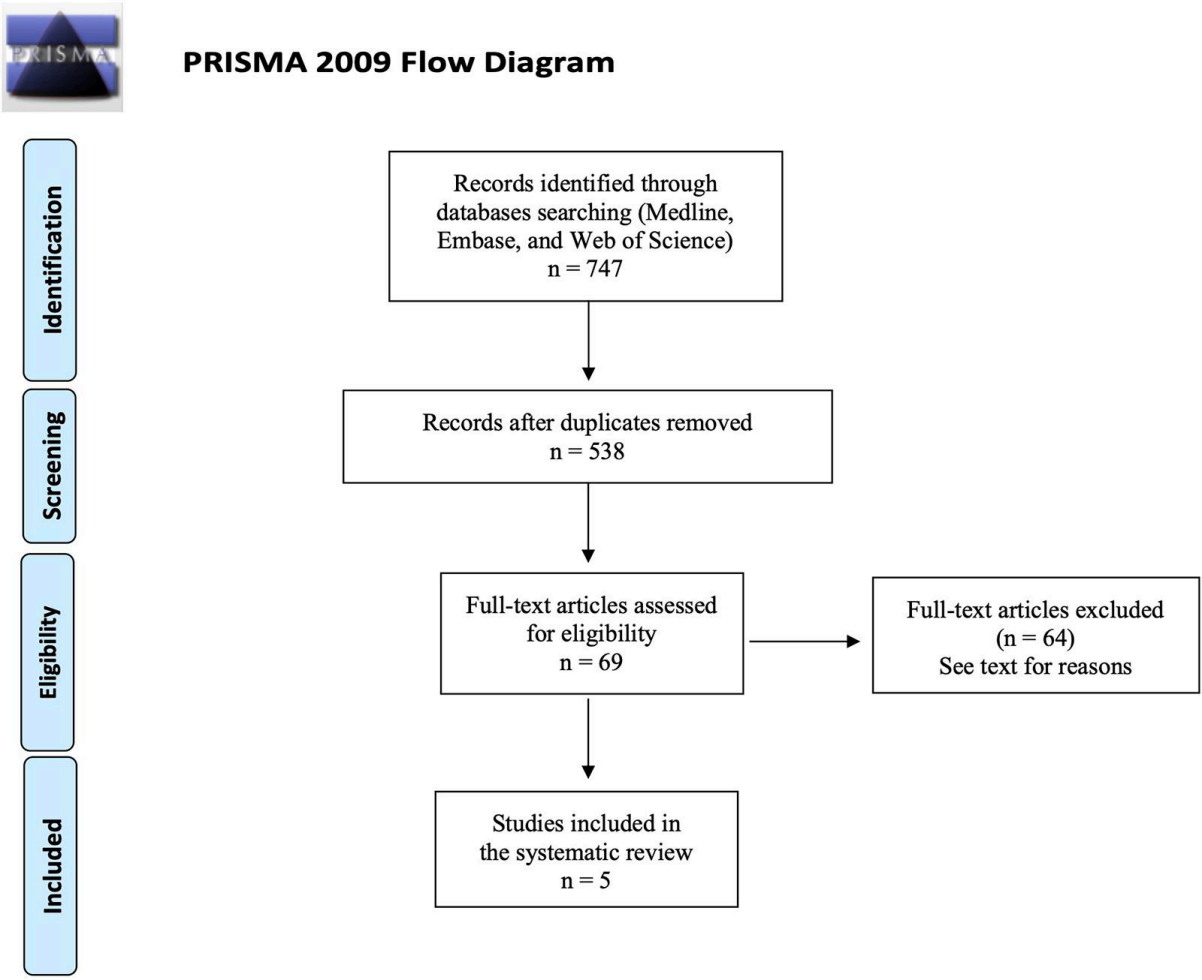
PRISMA flow diagram of included studies.

The initial search identified 747 records; after removal of duplicates, 538 titles and abstracts were assessed, and 69 full-text articles were examined. Of these, 64 were excluded because did not provide extractable oncologic outcomes, included only advanced or recurrent disease, or presented a study design incompatible with the predefined criteria. Five studies met all eligibility requirements and were ultimately included in this review **(**Figure 2**).**

### 4.1 Incidence and techniques

The incidence of PSM varies widely depending on study design, detection method, and institutional practices. Vergote et al. conducted a prospective observational study including 173 diagnostic laparoscopies performed in patients with advanced ovarian cancer. An open access technique with low-pressure pneumoperitoneum was used (9). The incidence of PSM was 17%, mainly detected on histological examination. Ramirez et al. reviewed 58 reported cases of PSM, of which 40 occurred in patients with ovarian cancer who mainly underwent diagnostic laparoscopy. The access technique was not consistently reported (10). Ataseven et al. performed a monocentric observational study including 250 laparoscopies, of which 214 underwent systematic port-site excision in patients with epithelial ovarian cancer. The laparoscopic technique was not standardized because many procedures were performed in external centers. The incidence of PSM was 46.7% (11) Zivanovic et al. analyzed 2,251 laparoscopic procedures performed in women with malignant disease, including 714 procedures for primary ovarian and tubal malignancies. An open Hasson technique was used for abdominal access. The overall incidence of PSM was 1.18%, increasing to 2.10% in patients with adnexal malignancies (12). Finally Lago et al. analyzed a retrospective cohort of 123 diagnostic laparoscopies performed in women with advanced ovarian cancer. An open access technique with midline trocar placement was used. PSM was detected in 49% of the resected port sites (13) (Table 1).

**Table 1:** Summary of Key findings from included studies.

Study	n (OC)	FIGO Stage Distribution	Histology Distribution	PSM Incidence	OS Impact	PSR Morbidity	Systematic PSR	Diagnostic Method
(10)	40	III–IV: 33; I–II: 7	N/A	N/A	N/A	Variable	Not applicable	N/A
(9)	173	IIIA: 2; IIIB: 2; IIIC: 119; IV: 50	Serous: 144; Other: 29	17%	No	Not reported	Partial (*n* = 71)	Histology + Clinical
(12)	682	N/A	N/A	2.2%	Reflects advanced disease	Not reported	No	Clinical + Imaging
(11)	214	IIIA-IIIC: 159; IA-IIB: 55	HGSOC: 158; Other: 56	46.7%	No	Yes (OR 2.68)	Yes	Histology
(13)	123	III: 104; IV: 16; N/A: 3	HGSOC: 90; Other: 33	49% (PSR group)	No	Yes (RR 2.42)	Partial	Histology

### 4.2 Risk factors

Advanced disease stage was consistently reported among patients with PSM. In the series by Ramirez et al., 83% of ovarian cancer patients with PSM had FIGO stage III–IV disease, 97% had carcinomatosis, and 71% presented with ascites at the time of laparoscopy (10). Vergote et al. included patients with advanced ovarian cancer almost exclusively, with 98% classified as FIGO stage IIIC–IV (9). Similarly, Ataseven et al. reported that 92.0% of PSM-positive patients had pT3c disease. The presence of large-volume ascites (>500 mL) was frequently documented. Ramirez et al. reported ascites in 71% of ovarian cancer patients with PSM (10). Ataseven et al. identified ascites >500 mL as a significant factor associated with PSM (OR 3.9, 95% CI 1.5–10.0, *p* = 0.005) (11). In the study by Lago et al., ascites >500 mL was present in 62% of patients (13).

As regards histology, high-grade serous ovarian carcinoma (HGSOC) was the predominant histological subtype among patients with PSM. Ataseven et al. reported HGSOC in 89.0% of PSM-positive patients (11). Lago et al. observed HGSOC in 73%–74% of patients, with no significant difference between PSM-positive and PSM-negative cases (*p* = 0.884) (13). Vergote et al. reported serous histology in 83% of the overall cohort. The interval between laparoscopy and detection of PSM varied widely across studies. Ramirez et al. reported a median time to diagnosis of 17 days (range 4–730) in ovarian cancer patients. Vergote et al. similarly observed that patients who developed trocar involvement had a median interval of 17 days, compared with 10 days in those without port-site disease, although this difference did not reach statistical significance (*p* = 0.09) (10). In contrast, Zivanovic et al. reported a median interval of 7 months from laparoscopy to diagnosis, with a wide range from 0.1 to 26.2 months (12). Institutional expertise also appeared to modulate risk. Ataseven et al. found that patients who underwent diagnostic laparoscopy in external, non-specialist centers had a markedly higher incidence of trocar-site metastasis (50.0% vs. 37.5%) in those treated at the specialist institution showing a strong trend toward significance (*p* = 0.04) (11).

### 4.3 Survival and morbidity

Ataseven et al. reported no statistically significant difference in overall survival (OS) between patients with and without PSM (HR 0.87, *p* = 0.642) (11). Lago et al. observed no survival differences between PSM-positive and PSM-negative patients (*p* = 0.92) (13). Vergote et al. reported a 3-year survival rate of 68% in patients with PSM compared with 37% in patients without PSM; this difference did not reach statistical significance (*p* = 0.12) (9). Zivanovic et al. reported a median OS of 19 months (95% CI 11.6–26.6) among patients with PSM highlighting that 95% had synchronous intraperitoneal or distant metastatic disease at the time of PSM diagnosis (12).

Port-site resection (PSR) was performed routinely in selected cohorts. Ataseven et al. reported PSR in 85.6% of patients undergoing laparoscopy prior to primary debulking surgery (11); no port-site recurrences were observed following excision. However, Clavien–Dindo grade 3–5 complications occurred in 41.0% of PSM-positive patients compared with 14.9% of PSM-negative patients (*p* < 0.001). Vergote et al. performed complete port-site excision in 71 patients, with histologically confirmed PSM identified in 31% of resected sites (9). Postoperative morbidity associated with PSR was reported in multiple studies: Lago et al. observed no survival differences between patients undergoing PSR and those without PSR (*p* = 0.28) but wound-related complications in 34% of patients undergoing PSR compared with 17% in the no-PSR group (RR 2.42, 95% CI 1.09–5.35, *p* = 0.029) (13).

## 5 Discussion

The present review is the first systematic review in the literature to address the clinically relevant issue of PSM in advanced ovarian cancer. However, it is based on a limited number of published studies, most of which are retrospective in nature, reflecting the scarcity of available data in the literature. Excluding the case series, the retrospective studies reviewed showed substantial variability in the incidence of PSM, ranging from 2% to 46%. These discrepancies highlight an essential distinction: clinically evident PSM are rare, whereas microscopic involvement of the trocar tract may be considerably more common. An important source of heterogeneity in the reported incidence of PSM is the variability in detection methods across studies, with some relying on clinical or imaging-based diagnosis and others on systematic histological assessment of resected port sites, the latter consistently identifying higher rates of microscopic disease. Across the available evidence, trocar-site metastasis appears to arise from a combination of disease-related and procedural factors rather than from a single causal pathway. Advanced intra-abdominal spread consistently emerged as the dominant determinant of risk, with all major series reporting higher rates of trocar implantation in women with stage III–IV disease (9–11). The presence of substantial ascites further amplified this susceptibility: large-volume peritoneal fluid, typically exceeding 500 milliliters, facilitated widespread exfoliation and dissemination of malignant cells, a relationship confirmed in two studies (11–13). Lymphatic involvement added another layer of risk, as positive nodal status independently predicted trocar metastasis in multivariable analyses (11). Procedural timing appeared relevant as well. Evidence drawn from clinical case series suggested that a prolonged interval between diagnostic laparoscopy and definitive cytoreduction may allow for implantation and early proliferation of displaced tumour cells (9, 10). Institutional expertise further modulated risk. Higher rates of trocar-site metastasis were reported among women whose diagnostic laparoscopy had been performed in non-specialist centres, implying that variations in laparoscopic technique, tissue handling, and perioperative management may influence the likelihood of tumour seeding (11).

Across all major series, trocar-site metastasis carried far less prognostic weight than its clinical visibility might suggest. Even in cohorts where abdominal wall involvement was relatively common, its presence did not independently worsen overall survival once disease stage, nodal status, and ascites were incorporated into multivariable models (11). Likewise, no survival difference emerged between women with or without histologically confirmed deposits at the time of cytoreduction (13). These findings indicate that trocar-site metastasis functions primarily as a surrogate of underlying tumour aggressiveness rather than an autonomous driver of prognosis. Its appearance reflects the extent and biological behaviour of peritoneal dissemination, while the clinical trajectory remains dictated by the systemic nature of the disease.

Several intraoperative strategies have been proposed to reduce the likelihood of tumour cell implantation, although none have proven definitively protective. Commonly recommended measures included controlled gas evacuation with the trocars still in place to minimize aerosolized cell escape (11, 13), careful fixation and limited manipulation of ports, and meticulous fascial closure of any incision larger than five millimeters, particularly in the presence of ascites (9, 10). Some authors also advised avoiding direct tumour biopsies when safer peritoneal sampling was feasible, as well as irrigating instruments and port sites, although consensus on these latter measures remains absent. Ultimately, even rigorous adherence to preventive techniques did not fully eliminate the risk of trocar implantation, as highlighted in the prospective observations of Vergote et al. (9).

Management strategies tended to favour excision of trocar sites at the time of primary or interval cytoreduction, with full-thickness removal of the abdominal wall from skin to peritoneum performed before intra-abdominal dissection. This approach was applied either routinely or selectively depending on the centre and operative conditions (9, 11). Separate fascial closure was standard. In the prospective cohort, no trocar-site recurrences occurred after excision, suggesting that local control can be confidently achieved when the abdominal wall tract is fully resected (9). However, this strategy carried measurable morbidity. In a direct comparison, wound complications were significantly more frequent among women undergoing PSR than in those whose trocar sites were left intact, with rates roughly doubling in the resection cohorts (11, 13). Complications included dehiscence, seroma, necrosis, and eventration, with higher-grade adverse events and longer hospitalization more common after excision. These findings underscore the need to balance potential preventive benefits against increased surgical burden.

These findings are based solely on extremely limited and heterogeneous retrospective case series, which substantially weakens any attempt to draw definitive or generalized conclusions. Further prospective studies and larger datasets are needed to better characterize this phenomenon and to clarify its clinical implications.

### 5.1 Management of giant PSM

Trocar-site metastases generally demonstrate an early onset and remain small in size, which typically allows for straightforward surgical management. However, cases of delayed giant PSMs, similar to the one presented here, have also been documented in the literature. Truly delayed PSMs presenting as large or giant abdominal wall masses are mostly described in case reports across different primary tumors. Chiva et al. reported a 73-year-old woman with uterine serous carcinoma who developed a 16 × 12 cm port-site recurrence in the right iliac fossa several months after laparoscopic staging and adjuvant radiotherapy; management consisted of palliative en-bloc resection with polypropylene mesh reconstruction followed by weekly paclitaxel plus bevacizumab (14). Greco et al. described an isolated PSM 2 years after laparoscopic partial nephrectomy for renal cell carcinoma, presenting as a 20 cm abdominal wall mass (21 × 13 × 11 cm, 1,180 g) that was completely excised with full-thickness abdominal wall resection; the patient remained disease-free at 1-year follow-up, showing that radical surgery can be curative when the lesion is truly solitary (15). Sharma and Chatterjee reported two late port-site recurrences 2–4 years after cholecystectomy in patients whose original specimens showed only chronic cholecystitis; both lesions were 4–6 cm abdominal wall masses compatible with gallbladder carcinoma metastasis, one with additional retroperitoneal nodal disease. Management combined surgical excision and gemcitabine–cisplatin chemotherapy, with 18F-FDG PET/CT used to confirm the diagnosis, exclude another primary and assess systemic spread (16). These data suggest that when the port-site lesion is solitary and completely resectable, aggressive en-bloc abdominal wall resection with mesh or flap reconstruction can achieve meaningful disease-free intervals, as illustrated by Greco et al. (15). Conversely, when giant port-site disease coexists with nodal or peritoneal metastases, as in the case from Chiva et al. (14). and one of the Sharma and Chatterjee patients (16), surgery is mainly palliative and overall prognosis is dictated by the extent and biology of the underlying malignancy. In the case we report, despite the application of all feasible preventive strategies, the patient developed a late-onset PSM which, not being surgically treated at initial recurrence (as it was not the only site of relapse), progressed despite second-line chemotherapy and ultimately reached a size that precluded any further surgical management.

### 5.2 The two-stage strategy

Several lines of evidence support the routine use of exploratory laparoscopy prior to primary cytoreductive surgery in advanced ovarian cancer, both from an oncologic and a health-economic perspective. From a survival standpoint, the two-stage strategy (diagnostic laparoscopy followed by delayed cytoreduction) does not appear to compromise patient outcomes. In a large multicenter cohort of FIGO III–IV ovarian cancer patients undergoing primary surgery, no significant differences in overall survival or recurrence-free survival were observed between one-stage and two-stage surgical sequences after adjustment for disease burden and surgical complexity, despite a short delay between procedures in the two-stage group. Even when cytoreduction was performed more than 15 days after diagnostic laparoscopy, survival outcomes remained comparable, suggesting that the interval required for adequate preoperative assessment does not translate into clinically meaningful harm (17). Beyond oncologic safety, the economic implications of preoperative laparoscopy are highly relevant. In a randomized controlled trial with an integrated cost-effectiveness analysis, diagnostic laparoscopy significantly reduced the rate of futile laparotomies without negatively affecting quality-adjusted life-years (QALYs). Although laparoscopy introduced additional upfront procedural costs, these were offset by savings related to avoided non-optimal cytoreductions, shorter hospital stays, and reduced postoperative care, resulting in overall cost neutrality and even a modest cost reduction. On a population level, treatment of 100 patients with a laparoscopic triage strategy was associated with a gain of one QALY and a net reduction in healthcare expenditure (18).

## 6 Conclusions

PSM following laparoscopy in advanced ovarian cancer are more frequent than previously assumed when histologically assessed. The occurrence of PSM is a source of considerable concern for clinicians, as it rekindles the debate regarding the safety of minimally invasive surgery in malignant disease. Insights from the present review indicate that, in advanced ovarian cancer, the development of PSM does not appear to adversely affect prognosis beyond the impact already conferred by the initial disease burden. The utility of routine PSR remains debatable due to associated morbidity, and its application should be tailored to individual patient risk profiles. Early detection is essential to ensure timely management and to minimize patient morbidity. Prospective studies are needed to standardize laparoscopic techniques and reduce the risk of PSM, particularly given the numerous advantages that minimally invasive surgery offers in this setting (19–21). Exploratory laparoscopy prior to debulking surgery is not only oncologically safe but also economically sound. By improving patient selection for primary cytoreduction and avoiding unnecessary extensive surgery (22), laparoscopy represents a rational and value-based component of the therapeutic pathway for advanced ovarian cancer, aligning optimal clinical outcomes with responsible resource utilization.

## Data Availability

The raw data supporting the conclusions of this article will be made available by the authors, without undue reservation.
